# The Molecular Landscape of Inflammation in Inflammatory Bowel Disease (IBD): Targets for Precision Medicine

**DOI:** 10.3390/biomedicines13112738

**Published:** 2025-11-09

**Authors:** Loris Riccardo Lopetuso, Marco Murgiano, Elisabetta Mantuano, Vincenzo Schiavone, Alessandro Costa, Gianluca Mascianà, Valentino Bezzerri, Gianluca Costa

**Affiliations:** 1 Department of Life Sciences, Health, and Health Professions, Link Campus University, 00165 Rome, Italy; l.lopetuso@unilink.it (L.R.L.); e.mantuano@unilink.it (E.M.); v.bezzerri@unilink.it (V.B.); 2 Medicina Interna e Gastroenterologia, CEMAD Centro Malattie dell’Apparato Digerente, Dipartimento di Scienze Mediche e Chirurgiche, Fondazione Policlinico Universitario Gemelli IRCCS, 00168 Rome, Italy; marcomurgiano1@gmail.com; 3 Dipartimento di Medicina e Chirurgia Traslazionale, Università Cattolica del Sacro Cuore, 00168 Rome, Italy; 4 Department of Advanced Biomedical Sciences, University of Naples “Federico II”, AOU “Federico II”, 80131 Naples, Italy; vincenzo.schiavone@unina.it; 5 UniCamillus School of Medicine, Saint Camillus International University of Health and Medical Sciences, 00131 Rome, Italy; u.005553@students.unicamillus.org; 6 Surgery Center, Colorectal Surgery Clinical and Research Unit, Fondazione Policlinico Universitario Campus Bio-Medico, University Campus Bio-Medico of Rome, 00128 Rome, Italy; g.masciana@policlinicocampus.it; 7 Cystic Fibrosis Center, Azienda Ospedaliera Universitaria Integrata, 37126 Verona, Italy

**Keywords:** inflammatory bowel disease, inflammation, crohn’s disease, ulcerative colitis, senescence, colorectal cancer, targeted therapy, oxidative stress

## Abstract

Inflammatory bowel diseases (IBDs), including Crohn’s disease (CD) and ulcerative colitis (UC), are chronic immune-mediated disorders characterized by mucosal injury, cycles of inflammation and repair, and tissue damage. Persistent inflammation accelerates epithelial turnover, generates oxidative and replication stress, and remodels the stromal niche, contributing to the risk of colorectal cancer (CRC). Systematic dysplasia surveillance remains essential. Cellular senescence has emerged as a unifying mechanism linking inflammation, impaired epithelial repair, fibrosis, and neoplasia. In UC, p16/p21 upregulation, telomere erosion, and loss of lamin B1 accumulate and adopt a senescence-associated secretory phenotype (SASP) that perpetuates barrier dysfunction. In CD, senescence within stem and stromal compartments limits regeneration, promotes pro-fibrotic remodeling, and sustains cycles of injury and repair via chronic SASP signaling. IBD prevalence continues to rise from environmental factors, dietary changes, antibiotic exposures, and gut microbiota alterations. Pathogenesis integrates genetic factors (e.g., NOD2, IL23R, HLA, and ATG16L1 mutations), environmental modifiers, dysbiosis characterized by loss of short-chain fatty-acid-producing Gram-positive bacteria and expansion of Proteobacteria, and a dysregulated immune system. Therapeutic strategies have shifted toward targeted biologics and small molecules to promote mucosal healing. In this review, we recapitulate the mechanistic axes of inflammation, oxidative stress, and senescence in IBD and then critically evaluate emerging targeted therapies. Topics include anti-TNFα, integrin blockade, IL-12/23 and IL-23 inhibition, JAK inhibitors, S1P receptor modulators, microRNA modulation, senomorphics, mesenchymal cell therapy, and microbiome interventions. We endorse biomarker-guided therapy and propose future directions to break the SASP-driven inflammatory loop and mitigate long-term carcinogenic risk.

## 1. Introduction

Inflammatory bowel disease (IBD) represents a spectrum of chronic, relapsing, immune-mediated disorders that predominantly afflict the gastrointestinal tract. The two major phenotypes, Crohn’s disease (CD) and ulcerative colitis (UC), differ in anatomical distribution, depth of inflammation, and complications but share overlapping pathogenic and clinical features. Despite remarkable advances in clinical and laboratory understanding of IBD, management remains challenging. Heterogeneous clinical phenotypes, an unpredictable disease course, and highly variable responses complicate therapy care. These difficulties are worsened by a steadily rising global prevalence, which was initially concentrated in Western nations but is currently increasing across South America, Asia, and the Middle East, likely due to urbanization, dietary and lifestyle shifts, widespread antibiotic exposure, and industrialization [1]. Current estimates place the global prevalence of IBD at almost seven million, with corresponding pressure on health systems [2]. This expansion reflects complex interactions between environment, diet, and host susceptibility [3]. Genetic predisposition is widely documented, with over 250 loci identified, many of which involve immune regulatory pathways. Notable instances include variations in NOD2, IL-23R, HLA, and ATG16L1 [4]. Environmental conditions are also major contributors [5]. Diets characterized by high intake of ultra-processed foods, refined sugars (including sugar-sweetened beverages), and poor-quality fats are associated with increased IBD risk, in particular CD [6,7,8]. Cigarette smoking exerts disease-specific effects. On the one hand, it increases the risk of CD. On the other hand, it seems to provide protection against UC, although with variation across populations [9]. Exposure to antibiotics in early life, urban living, and psychosocial stressors, such as anxiety and depression, significantly affect the onset and progression of disease [10,11]. Intestinal microbiota is known to have a major impact on the development of IBD. A few beneficial bacteria, like Firmicutes, are counterbalanced by many pro-inflammatory taxa, including Proteobacteria. This condition, known as dysbiosis, is associated with immune dysregulation, reduced synthesis of short-chain fatty acids, altered metabolism of bile acids, and impaired function of the intestinal barrier [12]. However, it is still unclear if dysbiosis causes or results from IBD. At the immunological level, disease-specific adaptive responses vary. Th1/Th17 pathways are primarily responsible for CD, while Th2 responses are linked to UC. TNF-α, IL-12, IL-23, IL-6, and IL-17 are cytokines that play a significant role in mediating the inflammatory cascade [13,14]. IBD is characterized by gastrointestinal symptoms, including lethargy, diarrhea, abdominal discomfort, and rectal bleeding. Nevertheless, dermatological illnesses (e.g., psoriasis, erythema nodosum, pyoderma gangrenosum), inflammatory eye diseases (e.g., uveitis, keratitis), psoriatic arthritis, enteropathic arthritis, and hepatobiliary disorders (i.e., primary sclerosing cholangitis) are other reported extra-intestinal features. The significant psychological impact of the illness is reflected in the prevalence of psychiatric comorbidities, including depression and anxiety [15]. Even with a better understanding of IBD, several challenges in managing IBD remain. These include loss of treatment effectiveness, lack of primary response to treatment, side effects, and complications. To improve care, it is crucial to adopt a personalized medicine approach that considers the unique genetic profile, microbiome, and immune system characteristics of each patient.

## 2. Interplay Among Inflammation, Oxidative Stress, and Senescence in IBD

Inflammation in IBD orchestrates a self-reinforcing loop involving pathological angiogenesis, oxidative stress, and cellular senescence. Pro-inflammatory cytokines and hypoxia drive pro-angiogenic programs (e.g., VEGF/angiopoietins), generating immature, leaky neo-vessels that amplify immune cell recruitment and mucosal edema. Microvascular dysfunction and hypoxia promote stress responses that heighten reactive oxygen and nitrogen species, intensifying oxidative DNA damage and replicative stress. In parallel, chronic cytokine signaling and oxidative injury promote epithelial and stromal senescence. The resulting SASP further induces angiogenic cues and redox imbalance, locking tissues into a cycle that sustains inflammation and primes dysplastic evolution.

The increased risk of colitis-associated colorectal cancer (CRC) is a critical long-term concern in IBD. The chronic mucosal inflammation is able to reshape epithelial biology, favoring a pro-tumorigenic milieu [16]. Chronic mucosal inflammation accelerates epithelial cell turnover and imposes oxidative and genotoxic stress on the intestinal lining through reactive oxygen (ROS) and nitrogen species (NOS), telomere erosion, and replication stress, which selects clones capable of surviving in an unfavorable environment. This partially explains why, despite modern therapies, long-standing, extensive colitis continues to confer a higher CRC risk than in the general population. Crucially, when dysplasia is detected early, CRC is highly treatable, underscoring the practical bridge between mechanism and clinical prevention. In recent years, cellular senescence has been implicated as a unifying process linking chronic injury, defective repair, fibrogenesis, and carcinogenesis across UC and CD. In UC, chronically inflamed mucosa frequently accumulates senescent epithelial cells, characterized by upregulation of p16 and p21 (cell cycle arrest markers), SA-β-gal activity, DNA damage with telomere shortening, loss of nuclear lamina components (e.g., lamin B1), and a pro-inflammatory SASP, which includes cytokines, chemokines, and proteases [17,18,19]. In the short term, senescence generally acts as a tumor-suppressive process by dampening the proliferation of damaged cells. However, SASP-driven cytokines, chemokines, and matrix-remodeling enzymes perpetuate inflammation over time, destabilizing barrier function and generating a niche in which dysplastic clones gain a competitive edge [20]. Computational and experimental profiling in UC increasingly identifies senescence-related gene signatures, including pathways such as LCN2-linked epithelial responses, that track with disease activity and dysplasia risk, highlighting how molecular programs of stress and repair can foreshadow neoplastic evolution [21]. In CD, senescence is often observed in epithelial stem and transient-amplifying compartments at lesion margins, where high levels of p16/p21 dampen regenerative capacity. The consequent failure to restore a healthy crypt architecture promotes persistence of lesions and recurrence in the same segments, while chronic SASP signaling fosters pro-fibrotic tissue remodeling [22]. Thus, the traditional clinical distinctions between UC and CD converge mechanistically on senescence-driven microenvironments that can both constrain and, in chronic conditions, facilitate malignant transformation. With chronicity, unresolved senescence and SASP sustain inflammation, remodel the extracellular matrix, perturb epithelial–mesenchymal cross-talk, and bias clonal dynamics toward dysplasia and carcinoma in both UC and colonic CD.

## 3. Targeted Therapies in Inflammatory Bowel Diseases

Over the past two decades, IBD therapy has progressively moved away from nonspecific immunosuppression toward more precise, mechanism-based interventions. Where once corticosteroids, azathioprine, and methotrexate were the mainstays, the current landscape is defined by biologics and small molecules designed to modulate discrete pathways. The core goals remain consistent: induce and maintain steroid-free remission, heal mucosa, prevent complications, and preserve long-term safety. In practice, the challenge lies in selecting the right agent for the right patient at the right time, given heterogeneity in disease behavior and therapeutic response. Below, we describe the classes of targeted therapies with mechanistic insight, comparative efficacy, and cautionary safety considerations, with particular emphasis on the newer agents in IBDs.

### 3.1. Anti-TNFα Agents

Despite remarkable advances in clinical and laboratory understanding of IBD, its management remains challenging. Heterogeneous clinical phenotypes, an unpredictable disease course, and highly variable responses complicate therapy care. The anti-TNF-α class remains the most widely used biologic therapy [23,24] and is still considered a first-line biologic option in both Crohn’s disease (CD) and ulcerative colitis (UC), according to contemporary ECCO guidance [25,26]. This class of drugs includes golimumab, infliximab, and adalimumab. These monoclonal antibodies neutralize TNF-α, a pro-inflammatory cytokine essential to IBD pathophysiology, by binding soluble and membrane-bound TNF-α, suppressing downstream inflammatory signaling, reducing pro-inflammatory cytokine release, and inducing apoptosis in activated T cells and macrophages [27,28,29]. Infliximab was tested for UC in the ACT1 and ACT2 clinical trials. After 8 weeks of treatment, approximately 65% of patients achieved a clinical response, with higher remission rates than placebo sustained at week 54 in the ACT1 study [30,31]. In CD, the ACCENT-I clinical trial showed that about 21% of patients undergoing infliximab treatment maintained remission at week 30 [32]. Adalimumab demonstrated long-term efficacy in CD in the CLASSIC/CHARM program, with remission rates around 40% at week 56 [33,34]. However, immunogenicity remains a significant issue. Almost 20–40% of patients may experience secondary loss of response related to anti-drug antibodies, for which dose optimization or switching to another agent is commonly employed [35,36,37]. Patients should also be monitored carefully for infection risk, including latent tuberculosis reactivation and other opportunistic infections, and screened and vaccinated in line with ECCO recommendations [38]. Particular caution and individualized risk-benefit discussions are advised for those with a history of malignancy [25,26].

### 3.2. Anti-Integrin Therapy

Vedolizumab is the prototypical gut-selective integrin blocker. It selectively binds α4β7 integrin on lymphocytes, preventing interaction with MAdCAM-1 on gut endothelial cells and thus attenuating lymphocyte homing specifically to the intestinal mucosa. Because it spares systemic cell trafficking, vedolizumab is relatively free from systemic immunosuppression [39]. This gut-selective immunomodulation limits systemic immunosuppression and lowers the risk of extraintestinal opportunistic effects. In UC, the GEMINI I trial demonstrated 42% remission at week 52 versus 16% on placebo [40], while in CD, the GEMINI II/III studies observed slower but meaningful induction and maintenance benefits over placebo [41]. The slower kinetics in CD are often attributed to the more transmural, patchy disease nature, requiring a longer time to alter the immune cell pool. In clinical practice, vedolizumab has shown consistent therapeutic outcomes in both UC and CD, with real-world data confirming remission rates of approximately 35–45% and mucosal healing in 30–40% of patients at one year [42,43]. Long-term extension studies demonstrated sustained clinical benefit, with over 80% of responders maintaining remission at 3- to 5-year follow-up [44]. Loss of response rates remains relatively low, and immunogenicity is rare, contributing to durable efficacy and favorable long-term safety profiles [45]. Vedolizumab’s favorable safety profile, minimal immunogenicity, and reduced systemic exposure make it especially attractive for patients at elevated infection risk or comorbidity burden, reinforcing vedolizumab’s role as a reliable and sustained therapeutic option in routine IBD management.

### 3.3. IL-12/23 and Selective IL-23 Blockade

The interleukin-23 (IL-23)/Th17 inflammatory axis has drawn increasing attention because of its central role in barrier immunity, mucosal inflammation, and autoimmune regulation. IL-23 blockade represents a paradigm shift from upstream targets (like TNF) toward more downstream and pathway-specific modulation. Ustekinumab, which binds the p40 subunit shared by IL-12 and IL-23, impedes both Th1- and Th17-driven inflammation. In CD (UNITI trials), up to 48–53% of treated patients achieved clinical remission at week 44, compared to 36% in the placebo [46,47]. In UC (UNIFI), approximately 38–44% of participants attained steroid-free remission at one year, versus 24% in controls [48]. Ustekinumab’s broader immunomodulatory effect preserves certain host-defense pathways (e.g., via IL-12) while attenuating pathogenic Th17 responses. Mirikizumab, a next-generation agent targeting the p19 subunit of IL-23, offers a more selective blockade by sparing IL-12–mediated pathways, potentially reducing infection risk and preserving anti-tumor immunosurveillance. In the phase 3 LUCENT 1/2 trials, mirikizumab achieved 24.2% clinical remission at week 12 versus 13.3% with placebo and maintained remission in 49.9% at week 52 compared to 25.1% with placebo (including secondary endpoints and safety outcomes) [48,49]. In a three-year open-label extension (extension of LUCENT-3), most induction responders maintained remission at 152 weeks without new safety signals, supporting the durability of effect. Real-world data from Japanese cohorts confirm short-term efficacy and safety, with approximately 44% remission at 12 weeks and decreases in C-reactive protein (CRP) and inflammatory biomarkers (without serious adverse events). Mechanistic correlative studies further illuminate mirikizumab’s action: mucosal gene-expression profiling in treated UC patients showed modulation of transcripts linked to anti-TNF or JAK-inhibitor resistance (e.g., IL1B, OSMR, FCGR3A/B, CXCL6), indicating that IL-23p19 blockade not only dampens inflammation but may reverse treatment-refractory gene programs. For patients who fail induction dosing, extended dosing strategies (600–1000 mg) yielded clinical response in up to 50% of nonresponders, many of whom maintained response through 52 weeks [47]. This suggests flexibility in optimizing dosing for difficult-to-treat phenotypes. Beyond UC, the IL-23p19 class is expanding into CD: risankizumab showed clinical remission in 35–45% at week 12 (versus ~25% placebo) in the ADVANCE and MOTIVATE trials [50]. Guselkumab, evaluated in the GALAXI-1 trial, achieved ~56% remission at week 12 (vs ~21% placebo), with sustained remission up to week 48 (~60–66%) [51]. In UC, the QUASAR trial reported durable, mostly steroid-free remission rates of ~72% at 92 weeks [52]. Collectively, the IL-23p19 inhibitors are carving out a central role for patients failing prior biologics or seeking lower immunogenicity and favorable safety in long-term therapy.

### 3.4. Jak Inhibitors

JAK inhibitors are small, orally available molecules that block downstream cytokine signaling. Although JAK inhibitors have a rapid onset and can be taken orally, patients must be closely monitored for lipid abnormalities, thromboembolic events, and infections, including herpes zoster. This class included Tofacitinib, Upadacitinib, and Filgotinib. Tofacitinib inhibits JAK1 and JAK3 (with partial activity on JAK2), broadly modulating multiple interleukin and interferon pathways [53]. It is currently approved for UC (but currently not CD). In the OCTAVE induction trials, remission rates of almost 18% at week 8 (vs 4–8% placebo) were demonstrated, and 34–40% of patients maintained remission at one year (many steroid-free). Its broad inhibition makes it powerful but less selective [54]. Upadacitinib is a selective JAK1 inhibitor, resulting in a more targeted blockade of pro-inflammatory cytokines, including IL-6, IL-12, IL-23, and IFN-γ [55]. It is approved for both UC and CD. In the U-ACHIEVE program, 26% remission at week 8 (vs 5% placebo) was achieved. Real-world studies report up to 65% steroid-free remission at 24 weeks. Meta-analyses of small molecules confirm superiority over placebo in both UC (clinical remission RR 3.16, endoscopic remission RR 3.99) and CD (clinical remission RR 1.53, endoscopic remission RR 4.78) [56]. Filgotinib also preferentially inhibits JAK1. It is currently approved for UC and under evaluation in CD. In SELECTION trials, remission rates at week 10 were almost 20% vs. 8% placebo, with sustained responses among responders [57].

### 3.5. Sphingosine-1-Phosphate (S1P) Receptor Modulators

S1P receptor modulators sequester lymphocytes in lymphoid tissues by inducing internalization and functional antagonism of S1P_1_ (and sometimes S1P_3/5_) receptors, thereby reducing lymphocyte egress and trafficking to inflamed sites. This mechanism differs from direct cytokine blockade and is suited to maintenance suppression. These are currently approved and used only for UC. This class included ozanimod and etrasimod. Ozanimod, an S1P_1/5_ agonist, in the TRUE NORTH trial, 18–21% of patients achieved remission at week 10 vs. 6% placebo. By week 52, almost 37% maintained remission vs. 18% in placebo arms. Meta-analyses place ozanimod’s relative risk for remission in UC at 2.52 and for endoscopic remission at 2.39, with additional histologic improvement (RR 2.20). Ozanimod does not require phosphorylation (unlike older S1P modulators) and appears to have a favorable cardiac safety margin; nonetheless, first-dose transient bradycardia, hepatic enzyme elevations, and macular edema must be monitored [58]. Etrasimod, a selective S1P_1/3/5_ agonist, was evaluated in the ELEVATE UC 52 trial, where 27% of patients achieved remission at week 12 (vs 7% placebo), and 32% maintained remission at week 52 (vs 7% placebo). Its safety profile is comparable to ozanimod in short-term studies. Indirect comparisons suggest broadly similar efficacy between the two agents, though head-to-head trials are lacking [59]. Because S1P modulators act on lymphocyte trafficking rather than cytokines per se, they may complement biologic or small-molecule anti-inflammatory strategies. Their oral administration and favorable utility in maintenance contexts make them a valuable addition, though long-term safety (infection, cardiac, hepatic) surveillance remains essential.

## 4. Emerging and Adjunctive Strategies

In recent years, new, promising therapeutic tools have emerged. Therapeutic strategies directed at microRNAs, especially miR-21 and miR-155, are being explored as a means to strengthen epithelial barrier integrity and restore immune regulation. Mesenchymal stem cell (MSC) therapy demonstrated effectiveness in inducing fistula healing in patients with refractory perianal CD. In UC, fecal microbiota transplantation has also shown promising outcomes. Current research is also looking into new cytokine pathways, metabolic modulators, and regulators of epithelial integrity. These new therapeutic approaches could lead to more and better ways to treat IBD.

### 4.1. Senotherapeutics and Senomorphics

Given the emerging centrality of cellular senescence in IBD pathobiology, therapeutic strategies that eliminate senescent cells (senolytics) or suppress their deleterious secretory phenotype without killing (senomorphics) are appealing.

Emerging senotherapeutics in IBD are biologically attractive because they target cellular senescence while potentially modulating the gut ecosystem, rather than broadly suppressing immunity. Drug repurposing of senomorphic molecules, including metformin, has been recently investigated. Metformin improved clinical signs and inflammatory markers in a randomized trial of mild-to-moderate UC [60]. In addition, mesenchymal stem cell therapy in UC cohorts has been associated with clinically relevant improvements alongside reductions in senescence-related markers, suggesting that the modulation of senescence programs could translate to patient benefit [61].

In preclinical colitis models, polyphenols such as fisetin can attenuate intestinal epithelial senescence markers (e.g., p53, p16, p21, and p38), suppressing SASP-related gene expression, including NF-κB-dependent cytokines and chemokines, STAT3, and COX-2, and favorably remodeling gut microbiota, supporting a mechanistic link between senescence modulation and barrier recovery [62]. Quercetin can exert pleiotropic effects, reinforcing tight junctions and mucus, rebalancing immune responses, and reshaping dysbiosis in the intestine [63]. In this context, a recent study revealed that quercetin-driven down-regulation of the cGAS–STING axis is able to restore M2/M1 macrophage polarity and barrier repair [64]. A prospective cohort study supported by in vivo experiments reported that higher habitual quercetin intake is associated with lower incidence of IBD and UC, aligning epidemiology with preclinical plausibility [65]. In small clinical studies, resveratrol improved disease activity and patient quality of life in UC, consistent with its antioxidant and NF-κB–inhibitory anti-inflammatory effects [66]. Among antioxidant vitamins, cholecalciferol (vitamin D3) supplementation raised serum 25(OH)D and was associated with reductions in UC disease activity in small randomized studies, while nano-formulations have shown additional benefit, strengthening the use of vitamin D for improving epithelial integrity and inflammation control [67]. Ascorbic acid (vitamin C) has a strong mechanistic rationale against ROS-driven epithelial and immune injury in IBD, though clinical translation remains poorly investigated [68]. N-acetylcysteine (NAC), a glutathione precursor, mitigates DSS colitis severity and histopathology in mice [69]. However, context-dependent pro-inflammatory effects of NAC have also been reported, underscoring the need for dose, timing, and disease-state stratification [70]. Targeting oxidative stress is a biologically credible adjunct to anti-inflammatory therapy in IBD, although heterogeneity across molecules and models argues for biomarker-guided studies that read out mucosal senescence, SASP, redox indices, microbiome shifts, and validated clinical endpoints.

### 4.2. MicroRNA Modulation

MicroRNAs (miRs) regulate post-transcriptional gene expression and are intimately linked to inflammation, barrier function, and epithelial homeostasis. miR-21 and miR-155, among others, have been implicated in IBD pathogenesis; antisense or mimic-based modulation may restore epithelial/immune balance or reduce SASP-driven loops. Though much of this work is preclinical or early-phase, the modularity and targeting specificity of miR-based approaches offer futuristic therapeutic promise [71].

### 4.3. Mesenchymal Stromal Cell Therapy

Mesenchymal stromal cells (MSCs) have immunomodulatory, anti-fibrotic, and regenerative properties. They have shown efficacy in complex perianal fistulizing CD and are under investigation in luminal disease. MSCs may suppress local inflammation, enhance repair, and modulate the local microenvironment to shift away from chronic injury. Challenges include cell sourcing, dosing, engraftment, and long-term safety [72].

### 4.4. Fecal Microbiota Transplantation and Microbiome Engineering

Fecal microbiota transplantation (FMT) has shown promise in inducing remission in UC, with meta-analyses supporting modest benefit. However, heterogeneity in donor selection, dosing, route, and recipient microbiome limits consistency. Engineered microbiota consortia, or precision bacteriotherapy, may ultimately offer more stable, mechanistically tailored interventions to restore barrier-friendly communities [73].

### 4.5. Combination and Dual-Targeted Therapy

In cases refractory to monotherapy, combining biologics (e.g., anti-TNF + vedolizumab) or overlapping mechanisms (e.g., biologic + small molecule) has been explored. Early studies of dual-targeted therapy suggest improved outcomes in refractory IBD or concomitant immune-mediated disease (IMID) without prohibitive safety issues, though controlled trials are needed. A 2025 meta-analysis of dual targeted therapy in refractory IBD found it to be effective and safe in many cases [74].

### 4.6. Biomarker-Guided Precision Therapy

As therapy options expand, the challenge is not only what to treat but also which patient with which mechanism at which time. Multimodal biomarker panels—including genomics, transcriptomics, mucosal senescent gene signatures, redox indices, microbiome signatures, and proteomics—are being evaluated to guide initial therapy selection, predict response or nonresponse, and enable safe de-escalation. For example, baseline mucosal oncostatin-M (OSM) overexpression has been repeatedly associated with primary nonresponse to anti-TNF in IBD. Embedding such biomarkers in clinical trials may shift decision-making from “trial-and-error” to more rational, individualized therapy paths [75].

## 5. Discussion

**Figure 1:**
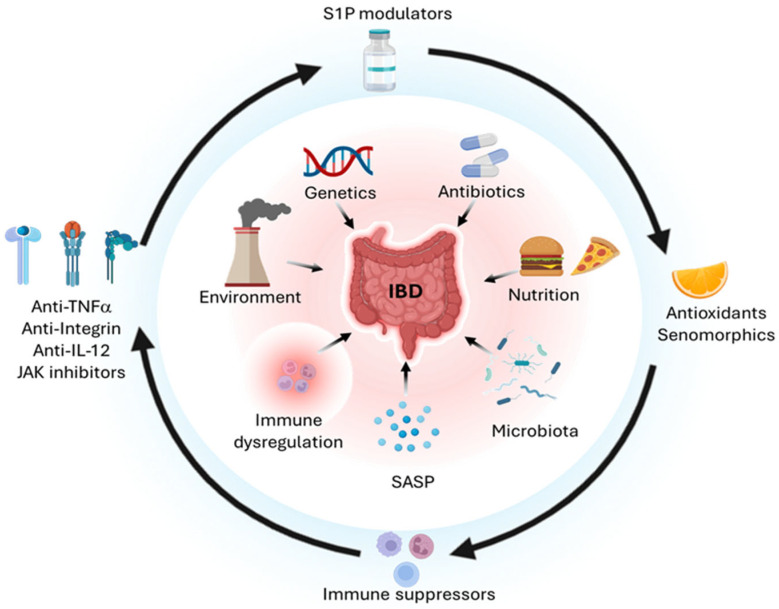
Pathogenetic mechanisms of IBD and targeted therapies. IBD arises from convergent environmental, genetic, drug-related, nutritional, microbiological, inflammatory, senescence-associated, and immunological drivers (inner circle). Corresponding targeted interventions, including anti-cytokines, JAK inhibitors, S1P modulators, antioxidants, senomorphics, and immunosuppressants, may exert beneficial effects on both the intestinal epithelium and the immune compartment (outer circle). Created in BioRender. Bezzerri, V. (2025) https://BioRender.com/4xmb2fm.

A central theme in IBD management is reducing the cumulative risk of colitis-associated CRC. Converging evidence supports the need for personalized medicine strategies based on early biomarker detection to reduce cumulative bowel damage, disability, and CRC risk [16,17]. Chronic inflammation, oxidative stress, and persistent SASP loops drive mutational burden and selective clonal outgrowth. Surveillance colonoscopy remains essential; in particular, high-resolution colonoscopy with dye-based chromoendoscopy may improve dysplasia detection over white light alone. In parallel, biomarker assessment (e.g., DNA methylation, microRNA panels, stool/serum markers) may augment surveillance sensitivity in the future [18,19,20,76]. Noninvasive markers such as fecal calprotectin (FC) and CRP provide fast, inexpensive readouts of mucosal inflammation that outperform symptoms for detecting active disease and for anticipating relapse or loss of response to therapies. Recent reviews and guideline updates consistently position FC/CRP as intermediate checkpoint indicators, underscoring that tightening control based on biomarker trends can shorten time to objective remission and lower the risk of flares or hospitalization [77,78]. Early and timely mucosal control (treat-to-target strategy) may help to control the neoplastic risk. In addition, precision de-escalation in deeply remitted patients, based on biomarker profiles, may reduce exposure to immunosuppression while maintaining control. The field is moving toward multimodal, predictive biomarker panels that pre-select the right mechanism for selecting patients within a precision medicine process. Integrative omics (i.e., genomic, transcriptomic, proteomic, metabolomic, and microbiome) and machine-learning models can identify baseline signatures associated with response or nonresponse to biologics and small molecules, although heterogeneity across studies argues for prospective standardization before clinical deployment [19,75]. Among candidate predictors, elevated OSM expression in mucosa or serum before therapy initiation has repeatedly been associated with primary nonresponse to anti-TNF therapy and use of rescue steroids in CD and UC. Nevertheless, FC remains a powerful dynamic predictor, reinforcing that novel biomarkers should complement rather than replace current biomarkers [77,78]. Early biomarker identification also improves cancer prevention, since chronic, smoldering inflammation reshapes epithelial biology and accelerates genotoxic stress. Timely suppression of mucosal inflammation may be chemopreventive, while structured surveillance is indispensable, with high-resolution colonoscopy, preferably with dye-based chromoendoscopy, improving dysplasia detection [79]. Blood or stool biomarkers are considered as complementary diagnostic tools to endoscopic inspection in long-standing colitis [80]. Within mechanism-guided therapy, anti-TNF agents remain feasible options in certain phenotypes, though immunogenicity and secondary loss of response mandate therapeutic drug monitoring and timely administration. Anti-integrin therapy (i.e., vedolizumab) offers durable control with favorable systemic safety, whereas IL-12/23 blockade (i.e., ustekinumab) and selective IL-23 inhibitors (risankizumab, mirikizumab) are increasing expectations, especially in CD [46,47,48,50]. Oral JAK inhibitors (e.g., Tofacitinib in UC and Upadacitinib in both UC and CD) are well suited to biomarker-driven tight control with appropriate monitoring for infections, lipids, and thromboembolic risk [53,54]. S1P receptor modulators (i.e., ozanimod and etrasimod) provide lymphocyte sequestration in the gut with maintenance of efficacy in UC [57,58]. Precision selection within the IL-23 blockers will likely be guided by baseline mucosal and blood omics signatures and microbiome configurations that can be rapidly and reproducibly assayed [46]. Embedding these signatures in clinical trials will be essential to demonstrate improved outcomes over canonical selection. Controlled de-escalation in patients with significant clinical, endoscopic, and histological remission will depend on biomarker signatures forecasting low relapse risk, enabling safer reduction in immunosuppression (Figure 1). Eventually, novel adjunctive strategies, including microbiome-modulating interventions, mesenchymal stem cell therapy [72], and senomorphics, are moving from bench research to bedside opportunities. Several studies, including systematic reviews, support MSC-based therapy as effective and safe for complex perianal fistulas [81,82,83], while fecal microbiota transplantation meta-analyses indicate potential for inducing remission in UC [73,84,85], although donor selection, dosing schedules, and delivery routes remain key limitations. Cellular senescence and SASP as a unifying axis linking chronic injury, impaired repair, and neoplasia invites exploration of specific biomarkers to improve risk stratification. Senotherapeutics, including senolytics and senomorphics, may provide targeted adjunctive strategies, even though these remain early translational approaches not yet ready for clinical development [86,87,88,89,90].

Standardized assays, harmonized cutoffs across platforms, and embedded biomarker-guided algorithms in real-world trials will ensure that any new signal adds actionable value beyond the already established markers FC and CRP. After rigorous validation and implementation, early biomarkers will improve timely, mechanism-matched therapeutic strategies that might change the natural history of IBD.

## 6. Conclusions

Over the past two decades, the therapeutic landscape in IBD has evolved dramatically. The transition from broad immunosuppression to targeted biologics and oral small molecules has improved remission rates and safety. The advent of IL-23p19 inhibitors, gut-selective integrin antagonists, JAK inhibitors, and S1P modulators offers a more nuanced arsenal. At the same time, emerging strategies, including senotherapeutics, microRNA modulation, MSCs, and microbiome therapy, are investigated to target complementary pathways. Critical for future clinical progress is embedding multimodal biomarkers into trials and clinical workflows, thus strengthening precision medicine approaches, timing, escalation, or de-escalation. Meanwhile, surveillance and cancer risk mitigation remain baseline imperatives. Ultimately, a paradigm combining mechanism-matched therapy with evolving adjunctive strategies holds promise to reshape the disease progression and reduce long-term complications.

## Data Availability

No original data were included in this Review.

## Abbreviations

The following abbreviations are used in this manuscript:

CD	Crohn’s disease
cGAS	Cyclic GMP-AMP synthase
COX-2	Cyclooxygenase-2
CRC	Colorectal cancer
IBD	Inflammatory bowel disease
IL-12	Interleukin-12
IL-17	Interleukin-17
IL-6	Interleukin-6
miR	Micro-RNA
MSC	Mesenchymal stromal cells
NAC	N-acetyl-cysteine
NF-kB	Nuclear factor kappa-light-chain-enhancer of activated B cells
NOS	Nitrogen species
ROS	Reactive oxygen species
SASP	Senescence-associated secretory phenotype
STAT3	Signal transducer and activator of transcription 3
STING	Stimulator of interferon genes
TNF-a	Tumor necrosis factor alpha
UC	Ulcerative colitis
